# Transcriptomic signatures of individual cell types in cerebral cavernous malformation

**DOI:** 10.1186/s12964-023-01301-2

**Published:** 2024-01-09

**Authors:** Ying Li, Romuald Girard, Abhinav Srinath, Diana Vera Cruz, Cezary Ciszewski, Chang Chen, Rhonda Lightle, Sharbel Romanos, Je Yeong Sone, Thomas Moore, Dorothy DeBiasse, Agnieszka Stadnik, Justine J. Lee, Robert Shenkar, Janne Koskimäki, Miguel A. Lopez-Ramirez, Douglas A. Marchuk, Mark H. Ginsberg, Mark L. Kahn, Changbin Shi, Issam A. Awad

**Affiliations:** 1 Department of Neurosurgery, First Affiliated Hospital of Harbin Medical University, Harbin, Heilongjiang, China; 2 Department of Neurological Surgery, Neurovascular Surgery Program, The University of Chicago, Chicago, IL USA; 3 Center for Research Informatics, The University of Chicago, Chicago, IL USA; 4 Human Disease and Immune Discovery Core, The University of Chicago, Chicago, IL USA; 5 Department of Neurosurgery, Division of Clinical Neurosciences, Turku University Hospital and University of Turku, Turku, Finland; 6 Department of Neurosurgery, Oulu University Hospital, Neurocenter, Oulu, Finland; 7 Department of Medicine, University of California, La Jolla, San Diego, CA USA; 8 Department of Pharmacology, University of California, La Jolla, San Diego, CA USA; 9 Department of Molecular Genetics and Microbiology, Duke University School of Medicine, Durham, NC USA; 10 Department of Medicine and Cardiovascular Institute, University of Pennsylvania, Philadelphia, PA USA; 11 Department of Neurological Surgery, University of Chicago Medicine, 5841 S Maryland, MC3026/Neurosurgery J341, Chicago, IL 60637 USA

**Keywords:** Cerebral cavernous malformation, VEGFA/VEGFR2, Transcriptome, Cell–cell interaction

## Abstract

Cerebral cavernous malformation (CCM) is a hemorrhagic neurovascular disease with no currently available therapeutics. Prior evidence suggests that different cell types may play a role in CCM pathogenesis. The contribution of each cell type to the dysfunctional cellular crosstalk remains unclear. Herein, RNA-seq was performed on fluorescence-activated cell sorted endothelial cells (ECs), pericytes, and neuroglia from CCM lesions and non-lesional brain tissue controls. Differentially Expressed Gene (DEG), pathway and Ligand-Receptor (LR) analyses were performed to characterize the dysfunctional genes of respective cell types within CCMs. Common DEGs among all three cell types were related to inflammation and endothelial-to-mesenchymal transition (EndMT). DEG and pathway analyses supported a role of lesional ECs in dysregulated angiogenesis and increased permeability. *VEGFA* was particularly upregulated in pericytes. Further pathway and LR analyses identified vascular endothelial growth factor A/ vascular endothelial growth factor receptor 2 signaling in lesional ECs and pericytes that would result in increased angiogenesis. Moreover, lesional pericytes and neuroglia predominantly showed DEGs and pathways mediating the immune response. Further analyses of cell specific gene alterations in CCM endorsed potential contribution to EndMT, coagulation, and a hypoxic microenvironment. Taken together, these findings motivate mechanistic hypotheses regarding non-endothelial contributions to lesion pathobiology and may lead to novel therapeutic targets.

Video Abstract

**Supplementary Information:**

The online version contains supplementary material available at 10.1186/s12964-023-01301-2.

## Introduction

Cerebral cavernous malformation (CCM) is a hemorrhagic neurovascular disease characterized by clusters of blood-filled capillary spaces lined by “leaky” endothelium [1, 2]. CCM patients present with highly variable symptomatology, including seizures, hemorrhagic activity, and focal neurologic deficits [2]. The chance of recurrent bleeding is tenfold higher, despite a low initial risk of hemorrhage estimated between 0.4% and 2.4% per year [2–4]. Currently, no medical treatment exists for this disease, while neurosurgical intervention presents with a high risk of morbidity, especially for brainstem and deep CCMs [1]. There are several ongoing clinical trials that are repurposing therapeutics to treat CCM, such as rho kinase inhibitors (atorvastatin, NCT02603328), reactive oxygen species (ROS) scavengers (REC-994, NCT05085561) and beta-blockers (propranolol, NCT03589014), but they remain in early phases of development [5–7].

CCM presents in either an autosomal dominant familial form, or a more common sporadic form [2]. Familial CCMs develop because of germ-line heterozygous loss of function (LOF) mutations in one of three CCM genes (*KRIT1*/*CCM1, Malcavernin/CCM2,* or *PDCD10*/*CCM3*) [8, 9]. Familial CCM lesions harbor biallelic endothelial cell (EC) mutations of the same CCM genes [10, 11]. Sporadic CCMs require either biallelic EC somatic LOF mutations of CCM genes, or a gain of function (GOF) somatic mutation of *MAP3K3* [12, 13]. Recent discoveries suggest that an additional GOF somatic mutation in *PIK3CA* may drive lesion development of both familial and sporadic CCMs [13, 14]. CCM proteins interact with a range of signaling processes, including cytoskeleton dynamics, angiogenesis, cell adhesion and migration, inflammation, and apoptosis [15–17]. Two preclinical studies recently showed that a loss of *Ccm3* in pericyte and neuronal cells led to the formation of CCM-like vascular malformations, suggesting a role of non-endothelial cells in CCM pathogenesis [18–20]. Another study using a *Ccm3* knockout mouse model reported that interactions between astrocytes and ECs drove CCM formation [21]. The results showed that ECs enhance production of nitric oxide (NO), which stabilizes Hypoxia-inducible factor (HIF)-1α in astrocytes, resulting in vascular endothelial growth factor (VEGF) overexpression and lesion formation [21]. We hypothesize that gene dysregulation in the CCM lesional milieu would likely reflect these cell–cell interactions and other cell specific contributions to the CCM lesion phenotype.

Previous transcriptomic studies of micro-dissected human CCM lesional neurovascular units identified differentially expressed genes (DEGs) related to angiogenesis, inflammation, junctional adhesion, apoptosis, and responses to oxidative stress [22]. Yet, the functional contribution of individual cell types in CCM lesions remains unclear. A comparison among the cell type specific differential transcriptomes would help clarify the contribution of individual cell types as well as dysfunctional cellular crosstalk that may be involved in the pathogenesis of CCMs.

## Methods

### Human tissue sample collection

Six CCM lesions were collected during surgical resection (3 sporadic/solitary and 3 familial/multifocal lesion) and four non-lesional control brain samples (3 from resection of non-lesional brain in epilepsy surgery and one involving normal brain resected in surgical approach to a sporadic CCM) (Table 1). Specimens were immediately embedded in optimal cutting temperature compound and snap-frozen in the operating room upon surgical resection, then stored at -80℃ until use.

**Table 1:** Demographics of CCM patients and non-lesional control

Sample	Age	Gender	Phenotype	Genotype
S1	11	Male	Familial/Multifocal CCM	CCM1
S2	2	Female	Familial/Multifocal CCM	CCM3
S3	38	Female	Familial/Multifocal CCM	Multifocal unknown genotype
S4	17	Male	Sporadic/Solitary CCM	N.A
S5	59	Male	Sporadic/Solitary CCM	N.A
S6	32	Female	Sporadic/Solitary CCM	N.A
C1	2	Male	Non-lesional Control	N.A
C2	4	Male	Non-lesional Control	N.A
C3	34	Male	Non-lesional Control	N.A
C4	43	Male	Non-lesional control	N.A

*N.A* Not Applicable

### Fluorescence-activated cell sorting (FACS)

Tissue was cut and minced on ice, and then enzymatically digested twice with 1 mg/ml Collagenase Type IV (MilliporeSigma, Darmstadt, Germany) and 100 μg/ml DNase I (MilliporeSigma) at 37℃ for 20 min and 30 min, respectively. The cell suspension was filtered, washed, and pelleted. Cells were then resuspended with 25% Percoll and underwent 20 min centrifugation without break. The top layer containing cell debris and myelin was removed. A multispectral LED light was used to perform a 30-min irradiation treatment to reduce background autofluorescence [23]. Cells were stained with an anti-human CD31-PE (303,105, BioLegend, San Diego, United States), CD45-BV421 (304,031, BioLegend), CD13-PE-Cy7 (301,711, BioLegend), P2RY12-FITC (392,107, BioLegend), CD49f-PerCP-Cy5.5 (313,617, BioLegend), CD90-BV711 (328,139, BioLegend) and GLAST-APC (130–123-555, Miltenyi Biotec, Bergisch Gladbach, Germany) antibody cocktail. Size, granularity, and antibody-specific gating were set to sort ECs, pericytes, microglia and neuroglia using the FACSymphony S6 Cell Sorter (BD Biosciences, Franklin Lakes, United States) (Fig. S1a).

### RNA extraction for FACS sorted cells

Individual cell types were sorted directly into 1 ml of TRIzol (Thermo Fisher Scientific, Waltham, United States) and lysed by pipetting several times. RNA was extracted using TRIzol in accordance with the manufacturer’s protocol with additional Phase Lock Gel-Heavy tubes used during the phase separation step [24]. RNA quantity and quality was determined using Bio-analyzer (Agilent, Santa Clara, United States). RNA concentration was 3.2 ± 3.3 ng/μl and RNA integrity number (RIN) was 2.46 ± 0.73. No difference was observed between the RIN values of CCMs and non-lesional brains.

### cDNA library preparation and RNA-seq

cDNA sequencing library preparations and sequencing were performed by the Genomics Facility at the University of Chicago using the SMARTer® Stranded Total RNA-Seq Kit-3-Pico Input (Takara, Shiga, Japan) and NovaSEQ 6000 sequencing system (Illumina, San Diego, United States). Two technical replicates per cell type and condition were generated. One of the FACS CCM EC failed the sequencing step. On average, 23.4 million bp single-end reads were generated per sample, from which an average of 3.64% was successfully aligned and used to produce counts per gene. Further analyses were performed on a random subset of 1 million pre-filtered, paired reads per sample, aligned to the complete human genome, to investigate the low percentage of mapped reads observed. The fractions of reads were aligned to the human genome reference GRCh38 using botwie2 (v 2.5.1) trimming 30 and 10 nucleotides of the 5' and the 3' ends respectively [25]. Finally, the fraction of reads mapped to canonical chromosomes from the reference were calculated. Results showed that most of the reads do not align to the human genome suggesting that there was neither genomic nor ribosomal contamination within the samples, and therefore are not likely to produce a bias on the counts.

### Differential gene expression analyses of single cell population

Sequencing data was processed using the RNAseq (v3.8.1) pipeline from the nf-core suite using as reference, the human genome GRCh38, gencode 34. A UMI-tools (v1.1.2) was used to remove PCR duplicates and salmon (v1.9.0) for alignment and quantification, generating the read counts per gene table. The quality of raw sequencing reads was assessed by FastQC (v0.11.9).

**Fig. 1:**
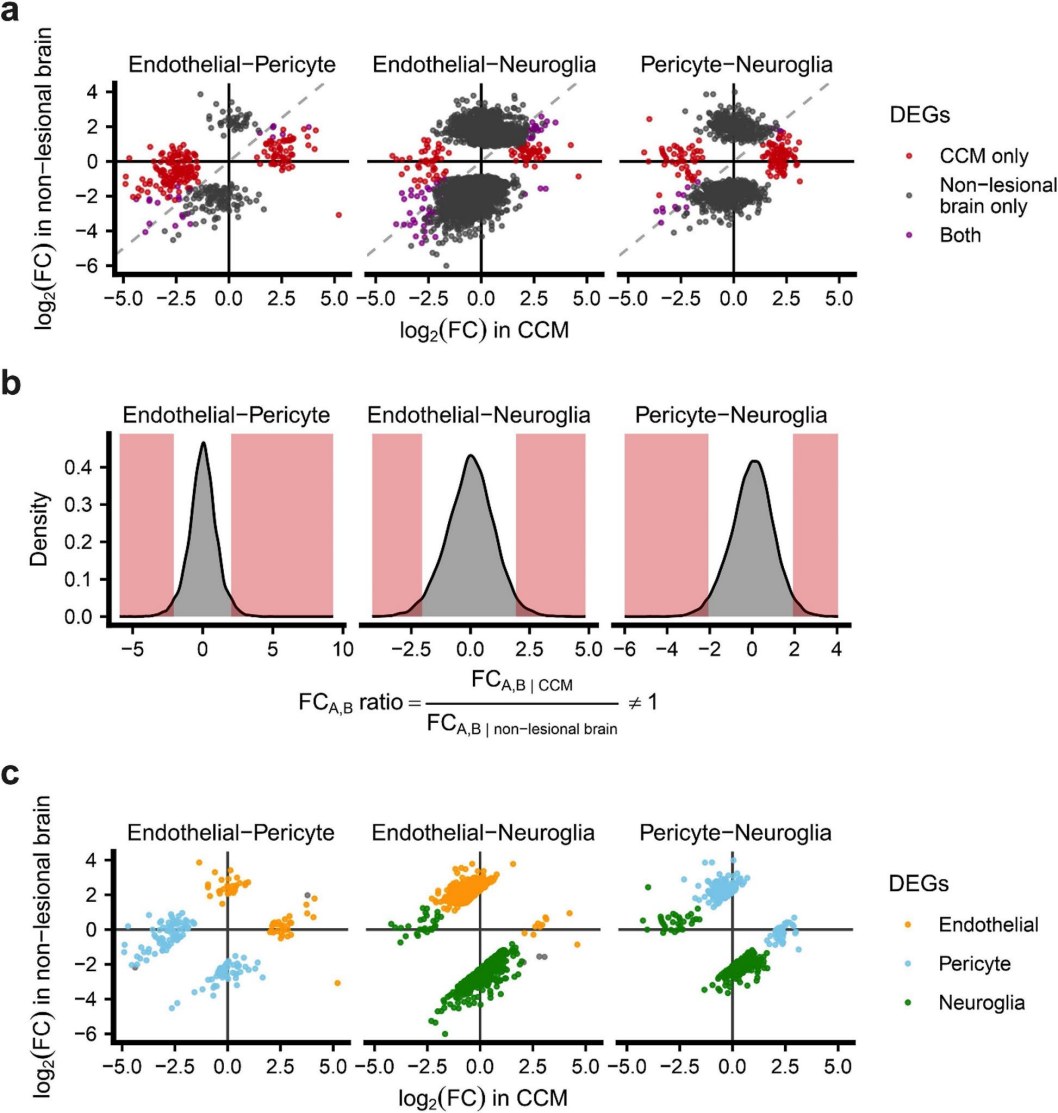
Expression of cell type-related genes is altered in CCMs after adjusting for cell type contribution. **A** Fold change of DEGs between two cell types in CCMs and/or non-lesional control brain. The red dots represent the DEGs identified only in CCM, the black only in non-lesional control brain while the purple in both. **B** z-score distribution of the FC_A,B_ ratio of two cell types. The red area shows higher difference in FC magnitude defined as FC_A,B_ ratio ≥ 1.96 standard deviation to the mean. **C** Altered genes in each cell type within CCM. The statistical significance of DEGs was *p* < 0.1, FDR corrected; |FC|> 1.5. The dots in orange indicate DEGs identified in endothelial cells, blue in pericytes and green in neuroglia. The list of DEGs is available in Table S4

DEG analyses (*p* < 0.1, false discovery rate [FDR] corrected; with absolute fold change [|FC|] > 1.5) were conducted using Limma (v3.52.0) in R (version 4.2.1), with an additive model for batch effect correction if necessary. Secondary analyses were further performed to identify cell-type-related genes altered in CCM. DEGs (*p* < 0.1, FDR corrected; |FC|> 1.5) were first identified and their FC calculated between two given cell populations (i.e., A and B) in (1) CCM and (2) non-lesional control (Fig. 1a). Differences in log_2_(FC) magnitude, defined as FC_A,B_ ratio, was then computed between CCM and control (Fig. 1b). A higher difference in log_2_(FC) magnitude was defined as a FC ratio greater than 1.96 SD to the mean (Fig. 1b).

### Pathway analyses

Ingenuity Pathway Analysis (IPA, Qiagen, Hilden, Germany) was performed to identify enriched canonical pathways (*p* < 0.01, FDR corrected). Non-CCM-disease-related pathways were excluded. Pathways with z-scores ≥ 2 are predicted to be activated and with z-scores ≤ -2 are predicted as inhibited [26]. IPA canonical pathways were then categorized into 5 biological processes, cellular proliferation, vascular processes, inflammation/immune response, apoptosis and oxidative stress, and permeability/adhesion, related to CCM disease based on the pathway description in the IPA database [26].

In addition, gene set enrichment analysis over the Hallmark gene sets from MSigDB, Gene Ontology (GO) enrichment and Kyoto Encyclopedia of Genes and Genomes (KEGG) pathway analyses (*p* < 0.01, FDR corrected) were also performed for each cell type using clusterProfiler R package (v4.6.2) [27].

### Ligand-receptor (LR) analyses

The CellChat package was adapted to identify the cell–cell interaction among ECs, pericytes and neuroglia. LR interaction analyses for single-cell RNA-seq are based on the observed expression per cell and the classification of cell populations. In this project, cells population were first sorted (ECs, pericytes or neuroglia) and validated by a list of gene markers with at least 70% sensitivity to identify a specific cell line using PanglaoDB (Supplemental Table 9) [28]. LR analyses were conducted via the CellChat package (https://github.com/sqjin/CellChat, v 1.6.1) in R (*p* < 0.05, FDR corrected) [29]. The communication probabilities of all LR interactions among three cell types in CCM and non-lesional control were calculated. Cell–cell communication architecture changes were investigated by projecting the inferred cell–cell communication networks onto a shared two-dimensional space based on similar signaling sources and targets. The difference of signaling networks related to certain LR pairs is defined as pathway distance, which was computed based on their Euclidean distance in the shared two-dimensional space. Larger distance implies larger difference in the communication networks between CCM and non-lesional control in terms of their functional similarity. *Refer to Supplemental Material for Supplemental Methods.*

## Results

### DEGs of each cell type of human CCMs compared to non-lesional controls

ECs, pericytes, neuroglia (defined as astrocytes and neurons) as well as microglia were sorted from six CCM lesions (3 sporadic CCMs and 3 familial CCMs) and four non-lesional control brain tissue (Fig. S1a). A list of gene markers showing over 70% of sensitivity in identifying a cell population was selected to validate FACS results (Table S1). The genes were then queried within the differential RNA seq profiling of each cell types against all the others (all: *p* < 0.1, FDR corrected; |FC|> 1.5). This validation approach confirmed the EC, pericyte and neuroglial populations (Fig. S1b-d), while it did not confirm microglia.

**Fig. 2:**
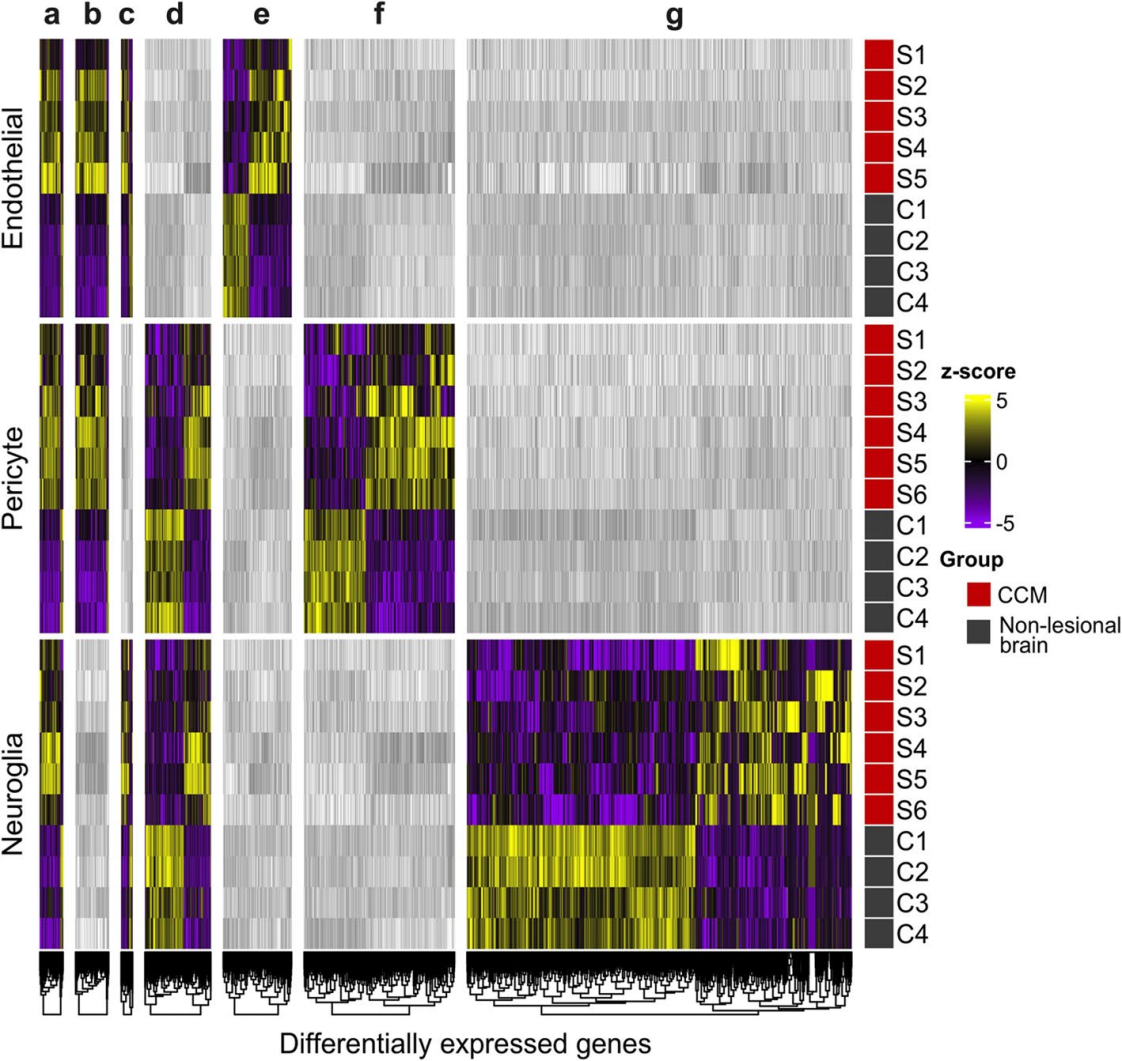
Heatmap showing the expression of DEGs identified in each cell type compared to their relative controls. **A** Ninety DEGs were common among the three cell types. **B** One hundred twenty-eight DEGs were in both endothelial cells and pericytes. **C** Forty-five DEGs were found both in endothelia and neuroglia. **D** Two hundred fifty-three DEGs were in both pericytes and neuroglia. **E**, **F** and **G** Two hundred sixty-three, 577 and 1473 DEGs were uniquely identified in endothelia, pericytes and neuroglia, respectively. The statistical significance of DEGs was *p* < 0.1, FDR corrected; |FC|> 1.5. The heatmap was calculated and presented based on z-score for normalized read counts. The colored scale bar on the right site indicates the color scaling with z-score values (yellow indicate significant upregulated genes; purple indicate significant downregulated genes; the gray scale of the genes represents the genes that were not identified as DEG in each group). The expression pattern of significant genes was grouped by similarity via hierarchical clustering analysis shown at the bottom of heatmap. The list of DEGs of each cell type is available in Table S2

Five hundred twenty-six DEGs were identified in ECs, 1048 in pericytes and 1861 in neuroglia isolated from CCMs compared to their respective human non-lesional brain control cells (*p* < 0.1, FDR corrected; with |FC|> 1.5) (Fig. 2 and Table S2).

Ninety DEGs were common among all cell types (Fig. 2a and Table S3a). Of interest, *SPI1*, *ADAM9*, *FNDC3B*, *LAMB1* and several genes coding for collagen proteins (*p* < 0.1, FDR corrected; |FC|> 1.5) were all upregulated in the 3 cell types. *HLA-DRB1*, *HLA-DRB5*, *C1QC*, *CFD*, and *TNFAIP2* (*p* < 0.1, FDR corrected; |FC|> 1.5), that are related to inflammation, were also upregulated. Taken together, these results support the ongoing extracellular matrix (ECM) remolding processes, which could induce endothelial-to-mesenchymal transition (EndMT), and local inflammatory microenvironment previously reported in CCMs [30–34].

The transcriptome of lesional ECs identified several upregulated genes that have been previously found dysregulated in CCMs, such as *RHOA, THBD*, *VWF*, *TGFB1, ANGPTL4* and *MSN* (*p* < 0.1, FDR corrected; |FC|> 1.5) (Table S2a) [31, 35–38]. These genes have been related to pathogenic mechanisms including endothelial proliferation, coagulation, cell adhesion and permeability [31, 35–38]. In addition, *PLVAP*, *SPARC* and *VIM* were also upregulated in ECs. An upregulation of *PLVAP* has previously been reported in pathological conditions associated with blood–brain barrier (BBB) dysfunction [39], while *SPARC, VIM* along with *TGFB1* and *MSN* are considered to be EndMT markers [40, 41].

Different cell types have unique gene expression signatures, and these may be altered in disease states [42, 43]. Secondary analyses were performed to identify the cell type-related genes whose expression is the most altered in CCM. For this analysis, the difference in log_2_(FC) magnitude of DEGs between two cell populations (*p* < 0.1, FDR corrected; |FC|> 1.5) was computed between CCM and non-lesional control (Fig. 1). Cell-type related genes altered under the disease state were defined as a higher difference in log_2_(FC) magnitude (≥ 1.96 standard deviation [SD] to the mean). The results of this secondary analysis showed a greater FC ratio of *THBD*, *VWF* and *PLVAP* suggesting these EC-related genes were altered in CCM (*p* < 0.1, FDR corrected; |FC|> 1.5) (Fig. 1 and Table S4). Of interest, it has been shown that thrombomodulin (*THBD*) was increased in both the lesion and plasma of CCM patients and is being tested as a potential biomarker for hemorrhagic risk in CCMs [37, 44].

The transcriptomic analyses of lesional pericytes further showed DEGs (*p* < 0.1, FDR corrected; |FC|> 1.5) associated with angiogenesis, cell adhesion, and EndMT, including *VEGFA, VCAM1, MMP2/9, FN1* as well as those related to antigen presentation (*HLA-DRB5, HLA-DPB1, HLA-DRA, HLA-DRB1, HLA-DQB1*) (Table S2b). Bulk sequencing of CCMs previously found these genes to be dysregulated but were not specifically associated with lesional pericytes [22, 31, 45]. In addition, *HSPA5* and *XBP1* were only upregulated in lesional pericytes. These genes have been related to endoplasmic reticulum (ER) stress that could lead to VEGFA production, triggering angiogenesis [46, 47]. The secondary analysis of log_2_(FC) magnitude identified *VEGFA, FN1,* and *MMP2/9* in lesional pericytes (Fig. 1 and Table S4).

Finally, the transcriptome analyses of the lesional neuroglia showed a dysregulation of *CD74, ADM, GBP2, CXCL8*/[IL8]*, IL6, CXCL1, CX3CL1, CXCR4, S100A8* and *VEGFA* (*p* < 0.1, FDR corrected; |FC|> 1.5) (Table S2c). These genes were previously shown to be involved in cell adhesion, inflammation, and angiogenesis in CCMs [22, 31]. Higher log_2_(FC) magnitudes were found of *S100A8, CXCL8* and *CXCL1* within lesional neuroglia (Fig. 1 and Table S4).

### Dysregulated pathways suggest functional contributions of each cell type within the CCM lesion

IPA and Hallmark gene set analysis via Gene Set Enrichment Analysis (GSEA) were further performed to assess the functional contribution and therefore gain insights into the functional working mechanisms of each cell type. Notably, IPA not only identifies the most significant pathways, but it can also predict the pathway status to be activated (z-scores ≥ 2) or inhibited (z-scores ≤ -2) [26].

**Fig. 3:**
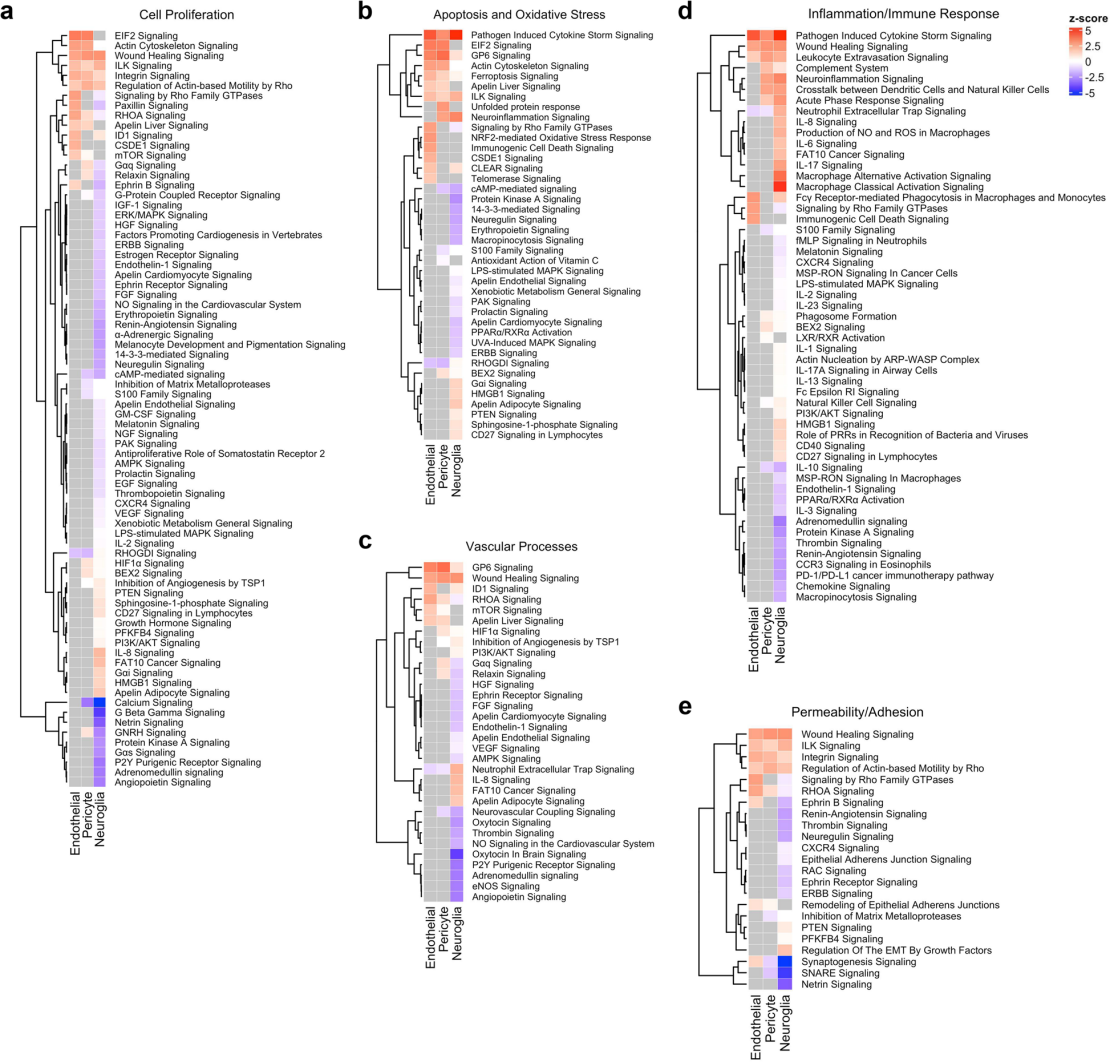
Enriched Ingenuity Pathway Analysis (IPA) pathways of each cell type were classified into 5 previously described categories based on their biological function. Five categories, defined based on previous reports, were (**A**) Cell Proliferation, (**B**) Apoptosis and Oxidative Stress, (**C**) Vascular Processes, (**D**) Inflammation/Immune Response, and (**E**) Permeability/Adhesion. The statistical significance of a pathway was defined as *p* < 0.01, FDR corrected. The colored scale bar on the right site indicated the color scaling with z-score values (red indicates activated pathway; blue indicates inhibited pathway; grey indicates no activity predictions can currently be made due to a lack of information in the Ingenuity Knowledge Base). The enriched pathways in each category were grouped by similarity via hierarchical clustering analysis showed at the left of heatmap. The list of IPA pathways is available in Table S5

Thirteen IPA pathways were enriched (*p* < 0.01, FDR corrected) based on the 90 DEGs common across all cell types (Table S3b). Further analyses showed that two IPA pathways, pathogen induced cytokine storm and wound healing signaling, were actually activated in all the cell types (*p* < 0.01, FDR corrected, z-score ≥ 2) (Fig. 3 and Table S5). The Hallmark gene set analysis also identified epithelial-mesenchymal-transition as enriched (*p* < 0.01, FDR corrected) (Table S3c). These results suggest that all three cell types are involved in the inflammatory response and EndMT processes.

**Fig. 4:**
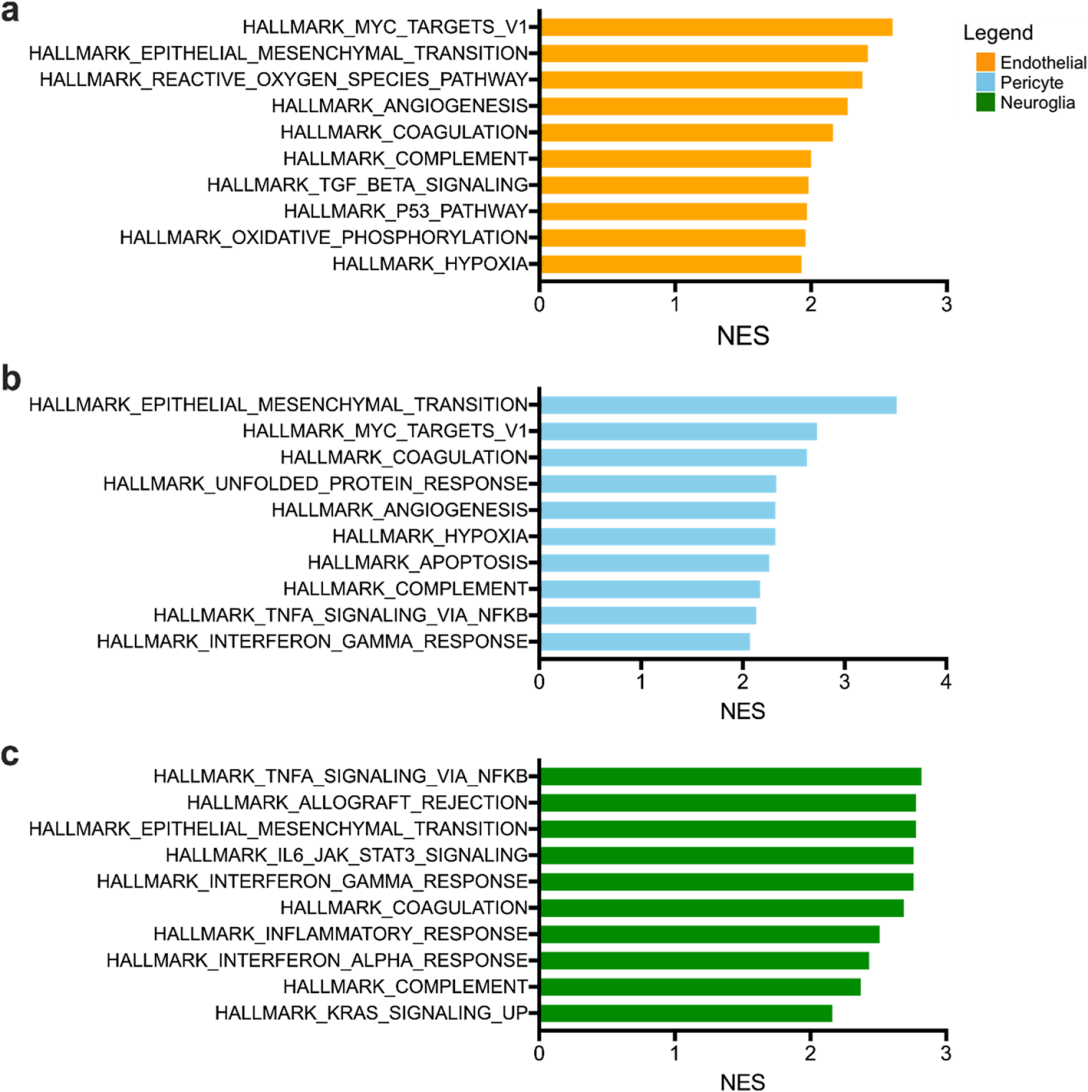
Top 10 Hallmark gene sets identified in each cell type. Hallmark Gene Set Enrichment Analysis (GSEA) was performed on gene expression data in each cell type, wherein genes were ranked by a weighted statistic for log_2_ (FC) and FDR corrected *p* value. The normalized enrichment scores (NES) of, at most, the top 10 enriched Hallmark gene sets were plotted for (**A**) endothelial cells, (**B**) pericytes, and (**C**) neuroglia. The statistical significance of Hallmark gene sets is *p* < 0.01, FDR corrected. The Hallmark GSEA data is available in Table S6

In lesional ECs specifically, 30 IPA canonical pathways (*p* < 0.01, FDR corrected) (Fig. 3 and Table S5a) and the top 10 Hallmark gene sets (*p* < 0.01, FDR corrected) (Fig. 4a and Table S6a) were mostly related to EC proliferation, EndMT, apoptosis and oxidative stress, angiogenesis, and coagulation. Of interest, GSEA identified Hallmark_Reactive_Oxygen_Species_Pathway only in lesional ECs (*p* < 0.01, FDR corrected) (Fig. 4a and Table S6a). Further IPA analyses showed that NRF2-mediated oxidative stress response, intrinsic prothrombin activation and immunogenic cell death signaling were activated (*p* < 0.01, FDR corrected, z-score ≥ 2) (Fig. 3 and Table S5a). ECs have actually been reported to be compromised by local oxidative stress and inflammatory stimuli, which act as key pathogenic factors of CCM development [22, 48].

Furthermore, 53 IPA pathways (*p* < 0.01, FDR corrected) (Fig. 3 and Table S5b) were identified in lesional pericytes and related to EndMT, cell proliferation, angiogenesis, coagulation, and inflammation, which was consistent with the top 10 Hallmark gene sets (*p* < 0.01, FDR corrected) (Fig. 4b, and Table S6b). ER stress pathway and unfolded protein response (UPR) were enriched and activated (*p* < 0.01, FDR corrected; z-score ≥ 2) only in CCM pericytes (Fig. 3 and Table S5b). The function of these two pathways has been associated with VEGFA production and angiogenesis [46]. In addition, antigen processing and presentation was identified as one of the top 5 GO terms and KEGG pathways (Table S7, Table S8). This suggests that pericytes may serve as non-traditional antigen presenting cells, contributing to the antigen-triggered immune response previously identified in CCM [32–34].

Finally, 148 IPA canonical pathways (*p* < 0.01, FDR corrected) were identified in neuroglia mostly related to neuroinflammation and EndMT (Fig. 3 and Table S5c). Five out of the 10 Hallmark gene sets identified were associated with inflammatory responses (*p* < 0.01, FDR corrected) (Fig. 4c and Table S6c). The production of NO and ROS in macrophages pathway was exclusively found to be activated in neuroglia (*p* < 0.01, FDR corrected) (Fig. 3 and Table S5c). We had previously shown that inflammatory DEGs predominate in mature CCM lesions, as compared to early-stage lesions in murine models [49]. Our current results suggested neuroglia are very likely involved in the inflammatory response observed in CCM [31].

### LR analyses among all three cell populations reveal distinctive cell–cell interactions in CCMs

Preclinical CCM studies suggest that non-cell-autonomous effects within cells composing lesional NVUs may occur during CCM pathogenesis. Signaling crosstalk via ligand and receptors among these cell types is critical for angiogenesis, EndMT, and inflammation all of which are related to CCM development [50–52].

**Fig. 5:**
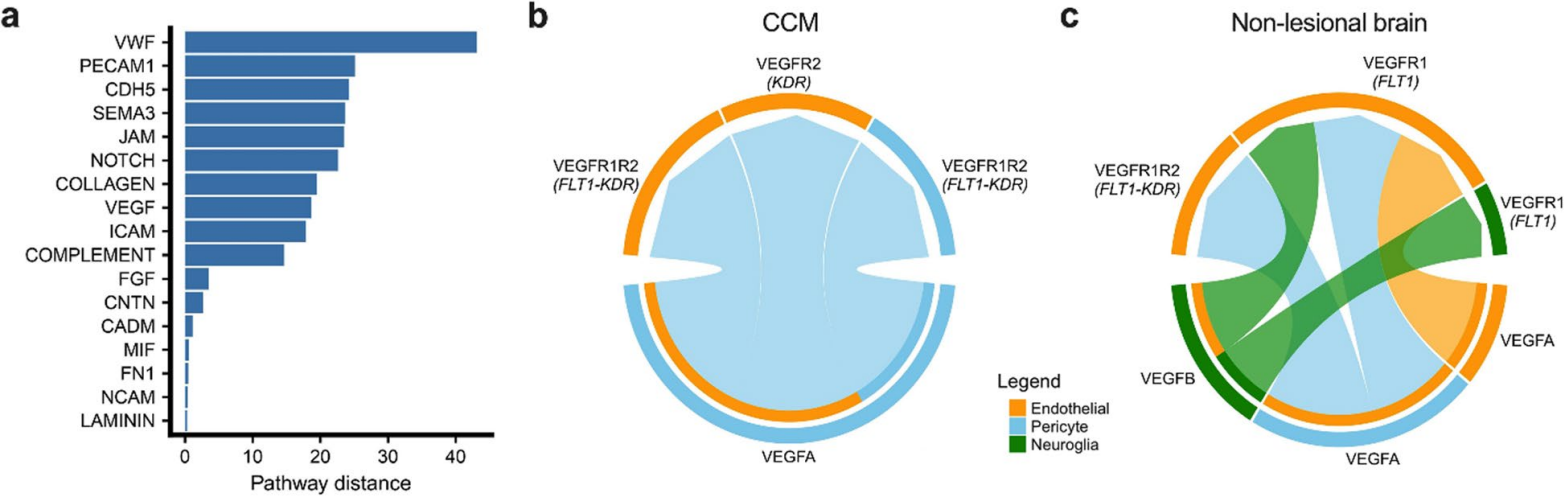
Ligand-Receptor (LR) analysis reveals specific cell–cell communications among individual cell types in CCM compared to non-lesional control brain. **A** The overlapping pathways were ranked based on their pairwise Euclidean distance in the shared 2-dimensional manifold. **B** Contribution of each LR interaction to the overall VEGF signaling in CCM. **C** Contribution of each LR interaction to the overall VEGF signaling in non-lesional control brain. The color bars of the inner semicircles indicate the target cell type of the outgoing signal (receptor). The significant results of LR interactions in B and C were defined as *p* < 0.05, FDR corrected. The list of ligand-receptor interactions is available in Table S9

The communication probabilities were calculated for all significant LR interactions to provide insight into how (1) these cell types interact and (2) such cell–cell interactions may contribute to disease, using CellChat (*p* < 0.05, FDR corrected) [29]. The differences of shared pathways including secreted signaling, ECM-receptor and cell–cell contact between CCMs and non-lesional control tissues were investigated by projecting the inferred cell–cell communication networks onto a shared two-dimensional space based on similar signaling sources and targets (Fig. 5a and Table S9).

**Fig. 6:**
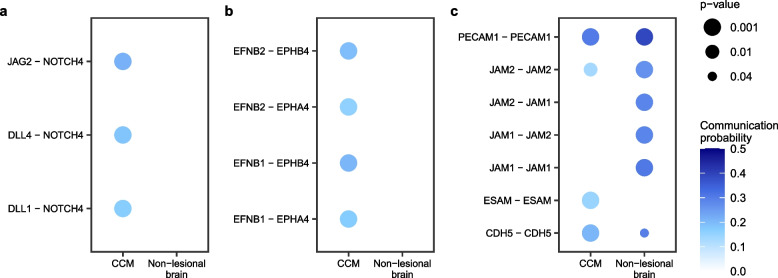
Ligand-Receptor (LR) interactions of the VEGFR2 downstream pathway between CCM lesional endothelial cells. These interactions include (**A**) NOTCH, (**B**) EphB4/EphrinB2 and (**C**) endothelial cell adhesion molecules. The significant results of LR interactions were defined as *p* < 0.05, FDR corrected. The list of ligand-receptor interactions among all the cell types is available in Table S9

Our results identified pathways related to angiogenesis (VEGF and NOTCH signaling), and endothelial cell–cell contact (platelet and endothelial cell adhesion molecule 1 [PECAM1], cadherin 5 [CDH5], and junctional adhesion molecule [JAM]) showed large functional difference between CCM and non-lesional control tissue (*p* < 0.05, FDR corrected) (Fig. 5a). The contribution of each LR interaction to the overall VEGF and NOTCH signaling in either CCM or non-lesional control was further identified and categorized (Fig. 5b, c; Fig. S2, and Table S9). The VEGFA-VEGF receptor (R)2(*KDR*) interaction was a distinctive LR interaction of VEGF signaling in CCM (Fig. 5b, and Table S9a). VEGF-NOTCH-EphrinB2 cascade plays an essential role in tumor vessel remodeling [53]. The interactions of NOTCH4 and its ligands, such as delta like canonical NOTCH ligand 1(DLL1), delta like canonical notch ligand 4 (DLL4) and jagged canonical NOTCH ligand 2 (JAG2), were identified in lesional ECs (Fig. 6a, Fig. S2, and Table S9). In addition, LR interactions of EphB signaling, a pathway important to regulation of VEGF and NOTCH signaling, was only identified in CCMs (Fig. 6b, and Table S9) [53, 54].

Previous studies have shown an increase in EC permeability due to loss of cell adhesion molecules between ECs in CCMs [9]. The LR analysis results showed interactions of cell adhesion molecules (KEGG: hsa04514) between ECs, such as CDH5, PECAM1, and JAM1/2, which were lower, whereas EC-selective adhesion molecule (ESAM) was higher in CCM (Fig. 6c and Table S9). Finally, no difference in LR interaction were identified between familial- and sporadic-CCM (*n* = 3 for each). *Refer to Supplemental Material for Supplemental Results.*

## Discussion

The goal of this study was to identify the transcriptomic signatures of cell types comprising the CCM lesion, and to infer potential cell–cell interactions. The pathway enrichment analyses of the 90 common DEGs among all 3 cell types, as expected, suggest local inflammatory response and EndMT [30]. In fact, *SPI1* has recently been identified as a novel, key driver of EndMT [45]. Numerous studies have suggested the inflammatory stimuli and EndMT processes within the CCM microenvironment are important to lesion pathogenesis [31–34]. Inflammatory cytokines have also been shown to be mediators during the coagulation process that may escalate thrombi formation and local hypoxic condition [31, 55, 56].

### Endothelial cells and pericytes are involved in angiogenesis through VEGFA-VEGFR2 signaling in CCM

Microthrombi within CCMs have been shown to induce local hypoxia leading to the activation of angiogenesis related genes [31, 56]. Hypoxia induces ER stress and stimulates the production of VEGF [46, 47]. IPA analyses suggested that ER stress pathway may be activated and lead to an UPR in lesional pericyte. ER stress can result in the accumulation of unfolded proteins, which binds to BiP (*HSPA5*), activating the UPR [46, 47]. Our results identified activation of *XBP1* in CCM lesional pericyte, a key modulator of UPR, could induce VEGFA production, independent of the HIF-1 pathway [46, 47]. VEGF, which is produced by pericytes, could be increased under hypoxic conditions [57, 58].

Additional LR analyses suggested that VEGFA-VEGFR2 interaction was only observed in lesional ECs and pericytes, while VEGFA-VEGFR1R2 interaction was identified in ECs and pericytes of both CCM and non-lesional controls. VEGFR1 is known to have a tenfold higher VEGF-binding affinity but tenfold lower kinase activity relative to VEGFR2 [59]. VEGFR1 may also serve to modulate VEGFR2 activation by antagonizing the VEGFR-2 responses [59]. VEGFR-2 mediates several key signaling processes involved in EC proliferation, migration, and survival [60].

**Fig. 7:**
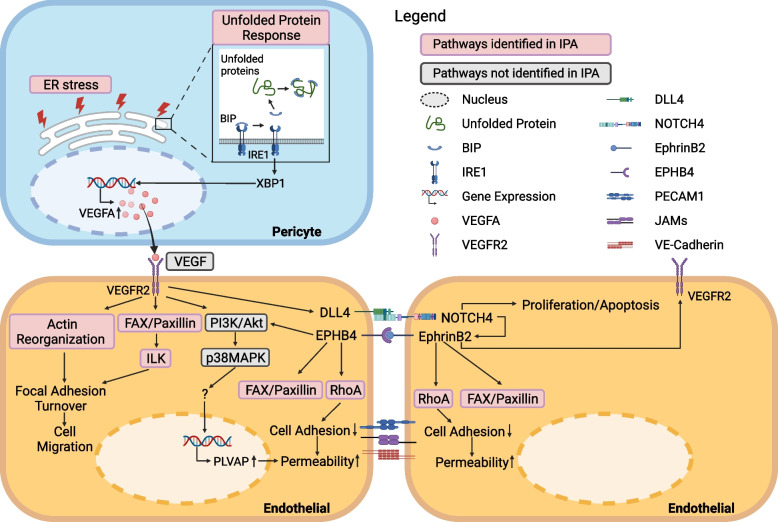
Hypothesized mechanisms of cell–cell communication between endothelial cells (ECs) and pericytes via VEGFA/VEGFR2 signaling in CCM lesions. Endoplasmic reticulum (ER) stress and unfolded protein response could induce VEGFA gene expression in pericytes. VEGFA from pericytes binds to VEGFR2 on ECs which leads to endothelial proliferation, migration, increasing permeability through complex intercellular and intracellular signaling

The results of the different analyses suggested that VEGFA signaling through VEGFR2 was the major pathway of angiogenesis between CCM lesional ECs and pericytes (Fig. 7). VEGFR2 could serve as a novel therapeutic target for CCM patients. Ramucirumab is a human monoclonal antibody directed against VEGFR2 approved by the FDA to treat different types of cancer [61]. A recent preclinical study showed that inhibition of VEGFR2 using SU5416 (Semaxanib), a specific VEGFR2 inhibitor, significantly decreased lesion burden in *Ccm1* endothelial-specific knockout mouse [62]. This suggests blockage of VEGFA/VEGFR2 signaling between endothelium and pericytes may prevent lesion growth.

Further analyses of processes downstream of VEGFR2 identified FAK-paxillin and ILK signaling activated in lesional ECs, which causes focal adhesion turnover and cell migration contributing to angiogenesis [63]. These processes can also be mediated through actin reorganization upon VEGFR2 activation [63].

*PLVAP* was upregulated in lesional ECs with a higher FC ratio compared to other cell types in CCM. This protein is associated with trans-endothelial transport and its overexpression in brain ECs only occurs in pathological conditions associated with compromised BBB function [39]. VEGFR2 signaling also induces Plasmalemma Vesicle Associated Protein (PLVAP) expression in a PI3K- or p38MAPK-dependent manner [39]. Recently, a single-cell study identified a unique PLVAP-positive EC subgroup with the PI3K/AKT/mTOR pathway strongly activated, even in non-mutant cells, in CCMs [45]. PLVAP could be targeted by novel nanocarriers, which may contain neutralizing antibodies, small molecule inhibitors, transcription inhibitors, or blockers, to achieve precise drug delivery [64].

Our LR analyses demonstrated drastic reduction of inter-EC adhesion molecule interactions in CCM lesions, consistent with increased permeability. In addition, ESAM interaction between ECs was only found in CCM. Its functions have been related to neutrophil extravasation, activation of Rho, and VEGF-induced vascular permeability [65]. The IPA analysis also identified pathways associated with ESAM in CCM lesional ECs, consistent with the LR results.

The LR interaction results showed DLL4/NOTCH-EphrinB2 (EFNB2) is identified only in CCM ECs. The ligand DLL4 can be upregulated by VEGF in the angiogenic vasculature and can activate NOTCH4- EFNB2 cascade in neighboring ECs [53, 63, 66]. Jag1, another NOTCH ligand, has opposing effects on angiogenesis [66]. Of interest, DLL4 is highly involved in directing endothelial sprouting, while Jag1 is required to achieve spatial control [67]. Furthermore, RhoA and paxillin signaling could be regulated by EphB4/EFNB2 signaling, which is involved in the regulation of the actin cytoskeleton, focal adhesion, and cell adhesion [54, 68]. This endorses hyperactive angiogenesis in CCM.

Taken together, our results motivate novel hypotheses for cell–cell interaction between ECs and pericytes in CCMs. VEGFA produced by pericytes, due to increasing ER stress and UPR, may interact with VEGFR2 in ECs to drive angiogenesis through complex intercellular and intracellular signaling (Fig. 7).

### Pericytes and neuroglia play a role in the inflammatory response

*VCAM1* and several genes that code major histocompatibility complex class II (MHC-II) were upregulated in lesional CCM pericytes. Pericytes have been shown to overexpress adhesion molecules such as VCAM1 to control immune cell trafficking across vessel walls [69]. Pericytes may exhibit macrophage-like activities such as phagocytosis, and act as antigen-presenting cells by displaying antigens through MHC-II [70]. Further analyses supported the antigen processing and presentation pathway in lesional CCM pericytes, strengthening its potential role in the neuroinflammatory response. Although there are DEGs and pathways related to cell adhesion and permeability, the primary function of neuroglia is inflammation. *CD74, ADM, CX3CL1* and *GBP2* are gene markers of reactive astrocyte [31], and were all upregulated in the CCM lesional neuroglia. Cytokines and chemokines, which are produced and released by reactive astrocytes, are necessary for leukocyte trafficking, and uptake of other inflammatory mediators [52].

Of interest, *S100A8/A9, IL6* and several TNF-α induced protein coding genes are overexpressed in neuroglia. S100A8, secreted from neurons under hypoxia, activates the secretion of TNF-α and IL-6 through the ERK pathway, which is mediated by TLR4 [71]. S100A8/A9 induces the secretion of various pro-inflammatory cytokines which are also necessary for recruitment of neutrophils and the production of ROS [72]. While these findings reflect previous studies on the immune response in CCM disease, they also highlight the importance of neuroglia as inflammatory mediators in CCM.

### Endothelial cells and pericytes contribute to EndMT and coagulation processes

EndMT markers, such as *SPARC, MSN, TGFB1*, and *VIM* were upregulated in lesional ECs [40, 41]. Inflammatory and angiogenic processes could initiate EndMT and cause ECs to lose cell junctions and migrate [35, 51]. Pericytes may arise by EndMT, which cancer studies have shown to potentially induce the abnormal recruitment and generation of pericyte-like cells to cover the vasculature [73]. However, gaps in pericyte coverage were found surrounding ECs in CCMs, due to significantly increased lesion vascular areas caused by proliferation and clonal expansion of mutated ECs [74, 75]. Our results reveal that *MMP2/9, FN1* and other DEGs related to type I/III collagen are overexpressed in lesional pericytes, suggesting involvement of pericytes in EndMT. During EndMT, the existing basement membrane is degraded by matrix metalloproteinases (MMPs), such as MMP-2 and MMP-9, and is replaced by new matrix molecules, including type I collagen, type III collagen and fibronectin [76]. It has been shown that increased fibronectin can cause capillary dilation, which may trigger CCM development [75, 77, 78].

Additionally, *THBD* and *VWF* were upregulated, and the intrinsic prothrombin activation pathway was identified as an activated pathway in CCM ECs. Thrombomodulin is a thrombin receptor present on EC membranes and thrombin has both pro- and anticoagulant functions [79]. Glycoproteins, like vWF and fibronectin, are exposed to circulating blood products, leading to platelet adhesion, activation, and aggregation to promote cell-based thrombin generation and blood coagulation when vascular integrity is disrupted [80, 81]. This may imply that the lesional ECs and pericytes are involved in immunothrombosis as validated in preclinical models [31].

### Limitations

It remains difficult to perform single-cell RNA sequencing on fresh-frozen tissue samples [82]. In addition, the mRNAs within the nuclei are less abundant and have higher heterogeneity therefore single-nuclei RNA sequencing lacks sensitivity to identify DEGs [83]. Finally, this study investigated common mechanisms of each cell type, regardless their genotypes, as well as their interactions. The scope of the study herein focuses on robust RNA seq analyses, and is therefore hypothesis generating. In addition, RNA-seq methods and data analysis approaches have been shown to be robust and not always require validation by qPCR and/or other approaches [84]. Additional orthologous validations of proposed mechanisms will however be performed in further studies.

A low fraction of reads mapping to the human genome were observed. The fraction of reads that mapped the human genome were within expected values. In addition, a low read count does not affect or bias the downstream analyses. Finally, a low-quality RNA and low mapping reads affect the sensitivity of detecting DEGs but not the specificity. Recent studies suggest that CCMs are a mosaic, including both mutant and non-mutant endothelium [74, 85]. Certain mutations other than CCM genes, such as *PIK3CA* and *MAP3K3,* have also been proven to contribute to CCM development [9, 86]. None of these known gene mutations related to CCM pathogenesis were identified as DEGs in our analyses of CCM lesional ECs. One explanation may be that the CCM samples used in this study included various genotypes, hence there was no predominance of individual EC mutations in DEG analyses. Cells were also sorted using FACS based on general surface marker regardless of gene mutations which they may harbor. Therefore, the signal of mutated cells in individual lesions were likely diluted by the more predominant non-mutated cells in all the lesions. In addition, there may be somatic mutations in non-ECs, that are currently not known. Nevertheless, the results herein support prior transcriptomic studies that identified pathogenic processes, such as inflammation, loss of focal/cell adhesion and increased vascular permeability that were common across different models of CCM disease regardless of their genotypes [22, 49].

Ren et al. (2023) recently reported a single-cell transcriptome atlas of most common GOF mutations identified in CCM ECs [45]. However, their study did not consider the non-autonomous effects observed in CCM milieu, particularly between mutant and non-mutants ECs. In addition, the effects we describe in this study proposes interactions between ECs with others lesional cell types, further strengthening their potential use as circulating biomarker. Future studies are currently being planned to clarify the DEGs in mutated versus non-mutated ECs in CCMs using spatial localization of mutated cells and their single-cell transcriptomics. Of interest, they showed that PLVAP-positive ECs contribute to VEGF signaling in CCMs [45]. Our study used a balanced cohort and the results not only confirmed these previous findings but also implied PLVAP may be one of key DEGs in CCM pathogenesis regardless their genotypes. The regulating interactions between *PLVAP* and *CCM* or other key regulators identified in CCM, such as KLF2/4, remain unknown. Future mechanistic studies may help to describe the potential interactions between *PLVAP* and *CCM* or other key regulators during CCM physiopathogenesis.

Neurons and astrocytes are considered to constitute the neuroglia population and were not separated by FACS in our protocols. Similar to previous findings, the expression level of VEGF is slightly higher in the CCM lesional neuroglia. But we could not comment on respective contributions of neurons or glia, and on whether the mixture of neurons and astrocytes may have blunted detection of that signal.

An LR analysis (CellChat) designed for single-cell transcriptome analysis was adapted to investigate the cell–cell communication [29]. CellChat seeks to infer potential LR interactions based on gene expression from annotated single-cell data with inferred cell types or groups of interest [29]. In this study, the single-cell annotation step was emulated by using transcriptomics of FACS cells labelled with specific surface markers. Each comparison involved 4 to 5 samples from each possible cell type pair in CCM or non-lesional control separately. While this implies a lower number of samples per group, this limitation is counteracted by a higher number of reads per gene than in single cell transcriptomics, and therefore reduced the sparsity in each sample. CellChat incorporates other important signaling cofactors, including soluble agonists, antagonists, as well as stimulatory and inhibitory co-receptors for a given LR interaction and adjusts for multiunit receptors accordingly [29]. This strict criterion results in a compendium of interactions with all the elements needed to occur in addition to actual spatial interaction between the cell types. However, certain receptors exhibit a stable and low mRNA expression level in cells, which may not be detected by RNA-seq [87]. Their activation status could also be interpreted by the altered activity of downstream transcription factors and gene regulatory networks. In addition, the type of LR interactions between the same cell type (self-self, subset to another independent subset of the same cell type) cannot be postulated, since the bulk expression per cell type was used. It is however reasonable to assume that most or some of the cells that belong to that cell type co-expressed all those LR components, but there is no guarantee that those are being co-expressed in the same cell. Nevertheless, results of LR analyses were congruent with the observations of DEGs and enriched pathways, giving additional support to these findings. The LR analyses did not identify difference between familial and sporadic CCM. This may be due to the low sample size (*n* = 3 in each group). There were not sufficient samples to assess unique signaling aberrations in different genotypes or in both sexes. Based on these initial discoveries, future studies will address these potential confounders.

We had previously published several studies on inflammatory cells in CCMs [32–34]. We did not address herein the differential transcriptome of CCM inflammatory cells, and we note that their control cells would not be readily available in non-lesional brain. Future study of differential transcriptomes of lesional versus peripheral inflammatory cells may add further insights about the nature of the pathogenetic inflammatory response in CCMs but is outside the scope of this project. In addition, further studies using spatial transcriptomic technology would also be necessary to analyze the changes in gene expression within lesional cells in the near microenvironment of CCMs.

## Conclusions

We suggest that one form of cell interaction between ECs and pericytes is through VEGFA/VEGFR2 signaling and leads to increased angiogenesis and vascular permeability in CCM disease. Furthermore, pericytes and neuroglia may mediate the immune response in CCMs. Finally, the results herein suggest that all the cell types are involved in EndMT and coagulation, which may reflect a hypoxic microenvironment that induces ER stress as well as VEGF signaling which were previously reported in CCM models [21, 62]. The results could motivate novel mechanistic hypotheses regarding non-EC contributions to lesion pathobiology and lead to new discovery of therapeutic targets.

### Supplementary Information

**Additional file 1:** Supplemental Methods, Supplemental Results, **Figure S1, Figure S2** and **Table S1.** 

**Additional file 2:** **Table S2 - Table S9.** 

## Data Availability

The raw sequencing data used in this study are available in the National Center for Biotechnology Information’s Gene Expression Omnibus (GEO) database and is accessible through GEO series accession number GSE233210.
