# Supplementary material for: Transcriptomic signatures of individual cell types in cerebral cavernous malformation
Source: Cell Commun Signal. 2024 Jan 9;22:23. doi: 10.1186/s12964-023-01301-2 (PMC10775676; doi:10.1186/s12964-023-01301-2)
Supplement: Supplementary file 2 — Additional file 1: Supplemental Methods, Supplemental Results, Figure S1, Figure S2 and Table S1. [file 12964_2023_1301_MOESM1_ESM.pdf]

Supplemental information

Transcriptomic signatures of individual cell types in cerebral cavernous malformation

Short title: Single-cell-type sequencing clarifies CCM mechanisms

Ying Li[1,2 †], Romuald Girard[2 †], Abhinav Srinath[2], Diana Vera Cruz[3], Cezary Ciszewski[4], Chang Chen[3], Rhonda Lightle[2], Sharbel Romanos[2], Je Yeong Sone[2], Thomas Moore[2], Dorothy DeBiasse[2], Agnieszka Stadnik[2], Justine J Lee[2], Robert Shenkar[2], Janne Koskimäki[5,6], Miguel A Lopez-Ramirez[7,8], Douglas A Marchuk[9], Mark H Ginsberg[7], Mark L Kahn[10], Changbin Shi[1*], Issam A. Awad[2*]

[1] Department of Neurosurgery, First Affiliated Hospital of Harbin Medical University, Harbin, Heilongjiang, China.

[2] Neurovascular Surgery Program, Department of Neurological Surgery, The University of Chicago, Chicago, IL, USA.

[3] Center for Research Informatics, The University of Chicago, Chicago, IL, USA.

[4] Human Disease and Immune Discovery Core, The University of Chicago, Chicago, IL, USA.

[5] Department of Neurosurgery, Division of Clinical Neurosciences, Turku University Hospital and University of Turku, Turku, Finland

[6] Department of Neurosurgery, Oulu University Hospital, Neurocenter, Oulu, Finland

[7] Department of Medicine, University of California, San Diego, La Jolla, CA, USA.

[8] Department of Pharmacology, University of California, San Diego, La Jolla, CA, USA.

[9] Department of Molecular Genetics and Microbiology, Duke University School of Medicine, Durham, NC, USA.

[10] Department of Medicine and Cardiovascular Institute, University of Pennsylvania, Philadelphia, PA, USA.

†Equal contribution as first authors

*Equal contribution as senior authors

Correspondence to: Issam A. Awad, MD, MSc, FACS, MA (hon), Department of Neurological Surgery, University of Chicago Medicine, 5841 S Maryland, MC3026/Neurosurgery J341, Chicago, IL, 60637. Phone: +1 773-702-2123, Fax +773-702-3518. Email: iawad@uchicago.edu.

Supplemental Methods

Sporadic and Familial diagnosis and genotyping

Familial CCM patients harbor multiple lesions throughout the brain on the most sensitive susceptibility weighted imaging (SWI) MRI sequence, a documented *CCM1*, *CCM2*, or *CCM3* germline mutation, and/or first-degree relative with a history of CCM. Familial case without genetic testing (e.g., deidentified sample from tissue biobank) was classified as *multifocal unknown genotype* [1].

Sporadic CCM cases harbored a solitary lesion on SWI MRI sequence, or a cluster of lesions associated with a developmental venous anomaly [1].

Validation of cell population from fluorescent cell activated sorting

A list of gene markers showing over 70% of sensitivity in identifying a cell population was first selected from PanglaoDB to validate fluorescent cell activated sorting (FACS) results (**Table S1**) [2]. Few specific genes markers have been identified in pericytes, therefore *CSPG4* (NG2), a well-known marker for pericytes, was also included [3,4]. The selected genes for each cell population were then confirmed to be cell-type specific using CellMarker 2.0 [5]. The genes were queried within the differential RNA seq profiling of each cell types against all the others regardless the tissue type (all: $p<0.1$, false discovery rate [FDR] corrected; with absolute fold change [|FC|]>1.5). The putative cell population were considered to be validated if the cell-specific gene expression markers were upregulated, when compared with all the other cell types.

Supplemental Results

An optimized FACS method purified four cell populations composing CCM lesional neurovascular units (NVUs)

Four distinct cell populations were sorted from 6 surgically resected lesions from cerebral cavernous malformation (CCM) patients (3 sporadic/solitary and 3 familial/multifocal lesion) and 4 non-lesional control brain tissues were processed (**Table 1**). Endothelial cells (ECs), pericytes, microglia, astrocytes, and neurons were labelled using specific cell surface markers. The cell populations were sorted using gating on size and granularity by forward versus side scatter (FSC vs SSC). The ECs ($CD31^+$, $CD13^-$, and $CD45^-$), pericytes ($CD13^+$, $CD31^-$, and $CD45^-$), neuroglia (including astrocyte and neuron, $GLAST^+$, $CD49f^+$, $CD24^+$, and $CD90^+$), and microglia ($P2RY12^+$, $CD45^+$) were independently sorted using the FACSymphony S6 cell sorter (**Fig. S1a**). Astrocytes and neurons were unable to be separated, due to the tissue preservation and preparation method.

Three sorted cell populations were validated by querying cell-specific gene expression markers in sequencing data

The validation approach of cell populations showed that *PECAM1* (CD31), *VWF*, *EGFL7*, and *CLDN5* ($p<0.1$, false discovery rate [FDR] corrected; with absolute fold change [|FC|]>1.5) were upregulated in ECs (**Fig. S1b and Table S1)**. In pericytes, *ANPEP* (CD13), *PDGFRB*, *NOTCH3*, and *CSPG4* (NG2) were upregulated (**Fig. S1c and Table S1)**. Finally, *SLC1A3* [GLAST1], *GFAP*, *S100B*, *AQP4*, *TUBB3*, *SNAP25*, *TMEM59L*, and *ENO2*) were upregulated in neuroglia (**Fig. S1d and Table S1)**. This validation approach confirmed the EC, pericyte and neuroglial populations, while did not support microglia.

Differentially Expressed Genes (DEGs) of each cell type in NVUs of human CCMs compared to non-lesional controls

One hundred twenty-eight DEGs were identified between ECs and pericytes, 45 between ECs and neuroglia, and 253 between pericytes and neuroglia ($p<0.1$, FDR corrected; $|FC|>1.5$) (**Fig. 2** and **Table S2**). Two hundred sixty-three, 577 and 1473 DEGs were only identified in endothelia, pericytes and neuroglia, respectively ($p<0.1$, FDR corrected; $|FC|>1.5$) (**Fig. 2** and **Table S2**).

The expression of cell type-related genes is altered in CCMs

The results of the secondary analyses on FC magnitude identified 111 genes between lesional ECs and pericytes (**Fig. 1** and **Table S4**). Of these 111 genes, 19 were previously identified as dysregulated in lesional ECs, and 80 in lesional pericytes (i.e., compared to their respective non-lesional control).

The comparison between ECs and neuroglia identified 42 genes (**Fig. 1** and **Table S4**), including 8 overlapped with DEGs in the lesional ECs, and 31 in lesional neuroglia (i.e., compared to their respective non-lesional control). Finally, the secondary analyses on FC magnitude identified 66 genes between lesional pericytes and neuroglia (**Fig. 1** and **Table S4**). Among these 66 genes, 22 were already defined as dysregulated in lesional pericytes and 39 in neuroglia (i.e., compared to their respective non-lesional control).

Enriched Gene Ontology (GO) term and Kyoto Encyclopedia of Genes and Genomes (KEGG) pathways support Ingenuity Pathway Analysis and Hallmark gene set results

Forty-five GO terms and 11 KEGG pathways (p<0.01, FDR corrected) were identified using the 90 common DEGs among all the cell type (**Tables S3d**, **e**). These data implied the microenvironment of CCM lesion including inflammation and coagulation and it might lead to endothelial-to-mesenchymal transition in lesion development.

Eighty-one GO terms and 26 KEGG pathways (p<0.01, FDR corrected) were found in lesional ECs, which indicated proliferation of ECs, as well as its involvement with focal and cell adhesion, interactions with extracellular matrix component, and coagulation (**Tables S7a** and **S8a**). There were 226 GO terms and 30 KEGG pathways (p<0.01, FDR corrected) identified in CCM pericyte. There results suggested the pericytes' role of antigen processing and presentation, focal and cell adhesion, and endoplasmic reticulum stress in CCM pathogenesis (**Tables S7b** and **S8b**). Five hundred sixty-seven GO terms and 112 KEGG pathways (p<0.01, FDR corrected) were identified in neuroglia within CCM lesion. These imply a neuroglia-mediated inflammatory response in CCM disease (**Tables S7c** and **S8c**).

Supplemental References

1. Akers A, Al-Shahi Salman R, I AA, Dahlem K, Flemming K, Hart B, Kim H, Jusue-Torres I, Kondziolka D, Lee C, Morrison L, Rigamonti D, Rebeiz T, Tournier-Lasserve E, Waggoner D, Whitehead K (2017) Synopsis of Guidelines for the Clinical Management of Cerebral Cavernous Malformations: Consensus Recommendations Based on Systematic Literature Review by the Angioma Alliance Scientific Advisory Board Clinical Experts Panel. Neurosurgery 80 (5):665-680. doi:10.1093/neuros/nyx091

2. Franzen O, Gan LM, Bjorkegren JLM (2019) PanglaoDB: a web server for exploration of mouse and human single-cell RNA sequencing data. Database (Oxford) 2019. doi:10.1093/database/baz046

3. Esteves CL, Donadeu FX (2018) Pericytes and their potential in regenerative medicine across species. Cytometry A 93 (1):50-59. doi:10.1002/cyto.a.23243

4. Stallcup WB (2018) The NG2 Proteoglycan in Pericyte Biology. Adv Exp Med Biol 1109:5-19. doi:10.1007/978-3-030-02601-1_2

5. Hu C, Li T, Xu Y, Zhang X, Li F, Bai J, Chen J, Jiang W, Yang K, Ou Q, Li X, Wang P, Zhang Y (2023) CellMarker 2.0: an updated database of manually curated cell markers in human/mouse and web tools based on scRNA-seq data. Nucleic Acids Res 51 (D1):D870-D876. doi:10.1093/nar/gkac947

Supplemental Figures

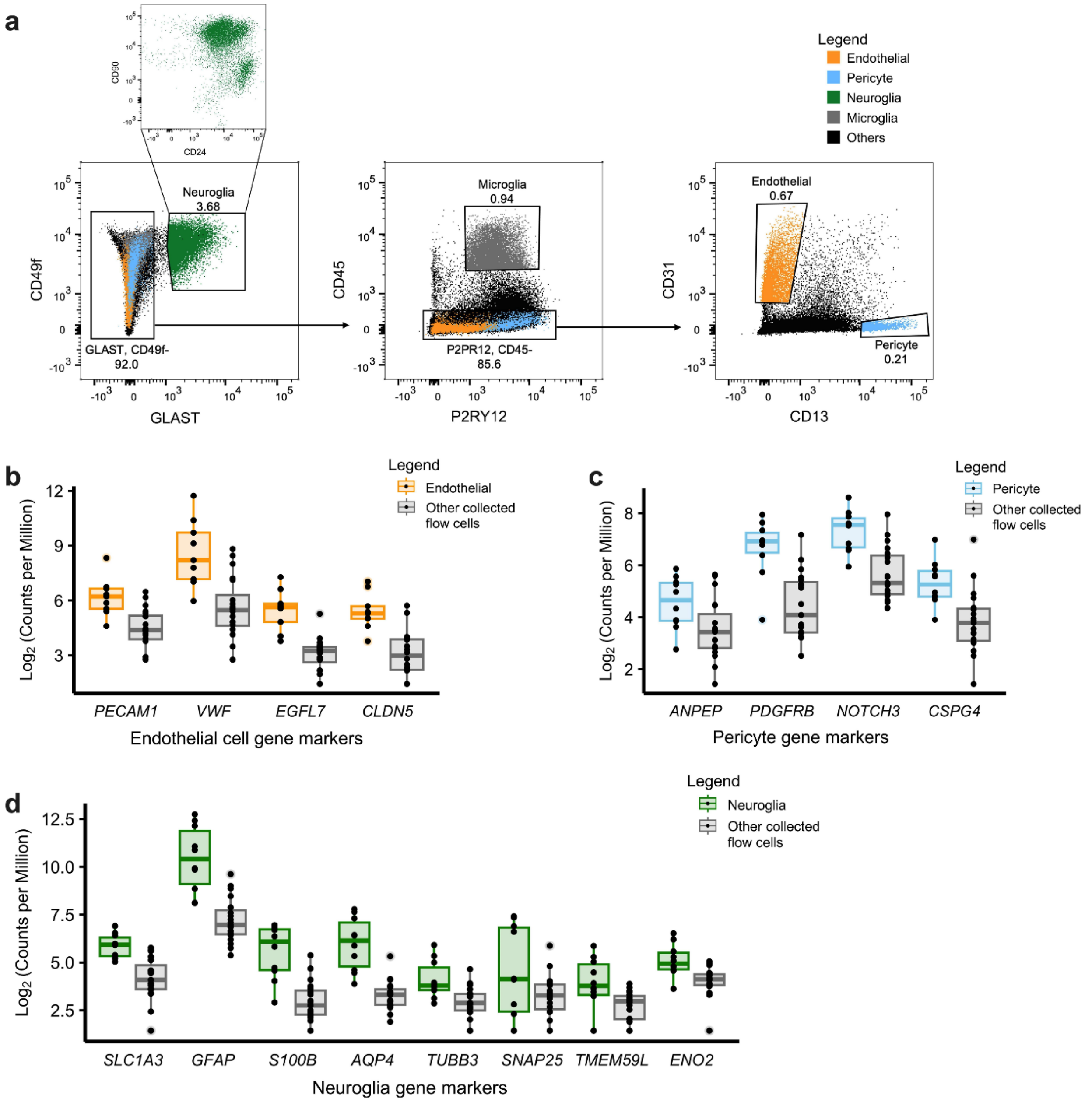

Fig. S1 Endothelial cells, pericytes and neuroglia were sorted and validated in all CCM lesions and non-lesional control brain tissues. (A) Gating of sorted cell population for RNA sequencing. (B), (C),and (D) Selected gene markers of endothelial cells, pericytes and neuroglia were queried in sequencing data. The statistical significance of gene markers is p<0.1, FDR corrected; with |FC| > 1.5.

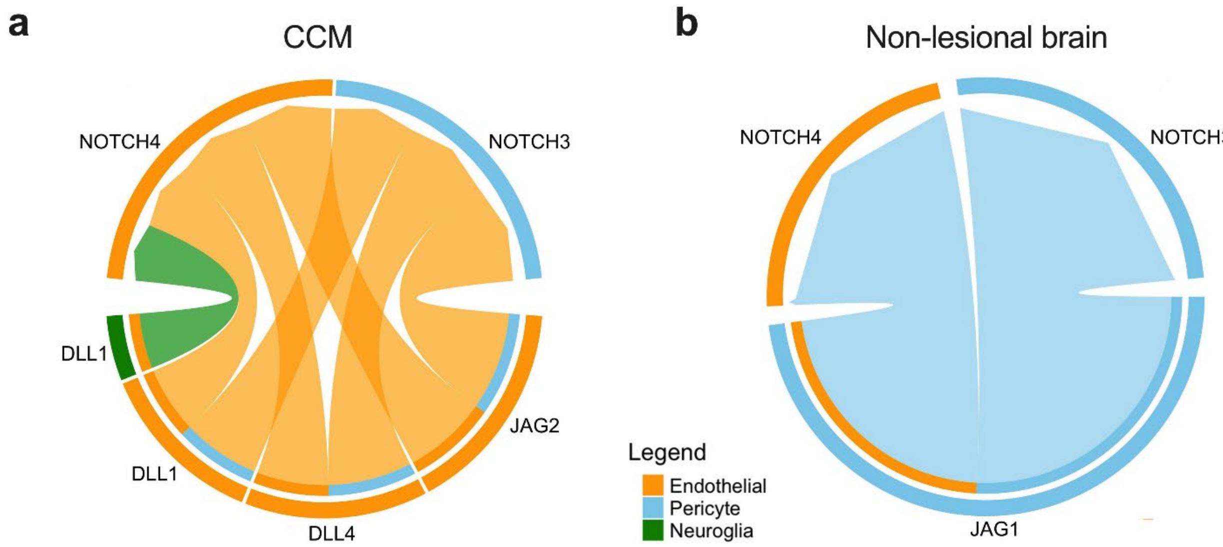

Fig. S2 Ligand-Receptor (LR) analysis revealed cell–cell communications via NOTCH signaling among individual cell types in CCM lesion compared to non-lesional control brain. (A) Contribution of each LR interaction of the overall NOTCH signaling in CCM lesion. (B) Contribution of each LR interaction of the overall NOTCH signaling in non-lesional control brain. The color bars of the inner semicircles indicate the target cell type of the outgoing signal (receptor). Ligand families depicted are Delta-Like ligands (DLL) and Jagged (JAG). The significant results of LR interaction were defined as $p<0.05$, FDR corrected. The list of LR interactions among all the cell types is available in **Supplemental Table 9**.

Table S1. Gene expression of selected gene markers of ECs, pericyte and neuroglia

Cell Type	Gene marker	$Log_2(FC)$	*p*-value	*p*-FDR corrected	Protein
Endothelial cell	*PECAM1*	1.75	7.12E-07	5.11E-04	CD31
	VWF	2.94	2.54E-07	2.33E-04	vWF
	EGFL7	2.79	2.85E-10	1.31E-06	VE-statin
	CLDN5	2.45	9.87E-08	1.06E-04	Claudin-5
Pericyte	*ANPEP*	1.50	1.00E-04	3.26E-02	CD13
	PDGFRB	2.79	3.85E-10	7.05E-06	PDGFRβ
	NOTCH3	2.27	2.66E-09	9.79E-06	NOTCH3
	GSPG4	1.64	1.78E-05	9.51E-03	NG2
Neuroglia	*SLC1A3*	1.87	7.97E-08	5.64E-05	GLAST
	GFAP	3.04	4.27E-07	1.60E-04	GFAP
	S100B	3.11	1.90E-09	7.64E-06	S100B
	AQP4	2.82	8.09E-10	4.94E-06	AQP4
	TUBB3	1.91	1.02E-05	1.47E-03	βIII-tubulin
	SNAP25	1.83	2.84E-03	4.68E-02	SNAP25
	TMEM59L	1.57	1.08E-04	6.27E-03	BSMAP
	ENO2	1.30	4.21E-05	3.59E-03	Enolase2
